# Ovarian Torsion in a 5-Year Old: A Case Report and Review

**DOI:** 10.1155/2012/679121

**Published:** 2012-06-07

**Authors:** Matthew F. Ryan, Bobby K. Desai

**Affiliations:** Department of Emergency Medicine, College of Medicine, University of Florida, P.O. Box 100186, Gainesville, FL 32610-0186, USA

## Abstract

Ovarian torsion represents a true surgical emergency. Prompt diagnosis is essential to ovarian salvage, and high clinical suspicion is important in this regard. Confounding the diagnosis in general are more commonly encountered abdominal complaints in the Emergency Department (ED) such as constipation, diarrhea, and urinary tract infections and more common surgical emergencies such as appendicitis. Prompt diagnosis can be further complicated in low-risk populations such as young children. Herein, we describe the case of a 5-year-old girl with a seemingly benign presentation of abdominal pain who was diagnosed in the ED and treated for acute ovarian torsion after two prior clinic visits. A brief discussion of evaluation, treatment, and management of ovarian torsion follows.

## 1. Introduction

Ovarian torsion can be an abdominal catastrophe for women, especially if ovarian salvage is not possible. Pathophysiological ramifications include ovarian loss, intra-abominal infection, sepsis, and even death. The psychological impacts can also be profound [1, 2]. Early diagnosis and high clinical suspicion are keys to prompt identification and definitive surgical treatment of this diagnostic dilemma. We report on a case of ovarian torsion in a five-year-old girl who initially presented to her pediatrician with nonspecific abdominal pain. There were several factors that confounded the diagnosis: firstly, was the fact that the patient had been seen and examined twice prior to presenting to the Emergency Department (ED). Secondly, the mistaken belief of the rarity of ovarian torsion within this patient population; five-year-old girls are well within the bimodal distribution for ovarian torsion. And lastly, the patient's seemingly benign presentation.

## 2. Case Report

A previously healthy five-year-old girl presented to the ED with a one-day history of fever and right lower quadrant pain. She had been evaluated by her pediatrician the previous day and was diagnosed with acute gastroenteritis after a benign physical exam and negative urinalysis. However, her symptoms progressed and her parents brought her to an after-hours urgent-care facility where a second urinalysis was performed and was again negative. The patient was again discharged with a diagnosis of gastroenteritis. The next morning the patient's pain had become more severe and she was sent to the ED by her pediatrician for further evaluation.

Upon presentation to the ED, the patient appeared ill and uncomfortable. She complained of constant pain not relieved with acetaminophen or ibuprofen. Her review of symptoms was positive for fever, one episode of vomiting, and abdominal pain. She was born full-term and has a history of asthma managed with inhaled corticosteroids and albuterol. She has two siblings who were healthy, and she had no ill contacts.

On physical exam, the patient's vital signs were within normal parameters for her age. Her oral temperature was 36.5°C, heart rate of 90, blood pressure of 110/70, and respiratory rate of 18, and oxygen saturation was 100% on room air. The patient's abdomen was slightly distended and tender in the right lower quadrant, pelvic, and suprapubic areas. She had some involuntary guarding but no rebound tenderness, hepatosplenomegaly, or costovertebral angle tenderness. Pain was elicited specifically at the right inguinal ligament. Classic signs for appendicitis, including Rovsing's and Psoas signs, were absent. The remainder of the exam was normal including heart tones, lung sounds, capillary refill, and skin turgor.

Her catheterized urine sample showed no protein, ketones, nitrites, or leukocyte esterase; the microscopic UA showed 30–50 red blood cells with no white cells or bacteria. The patient's white blood count was elevated at 14.6 thou/mm^3^ while hemoglobin, hematocrit, and platelets were within normal levels. A basic chemistry panel was normal.

**Figure 1:**
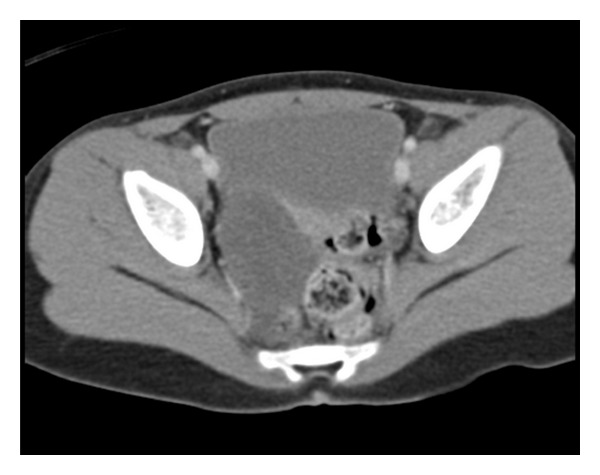
Computed tomography of the pelvis with IV contrast demonstrates a complex loculated fluid collection within right adnexa.

**Figure 2:**
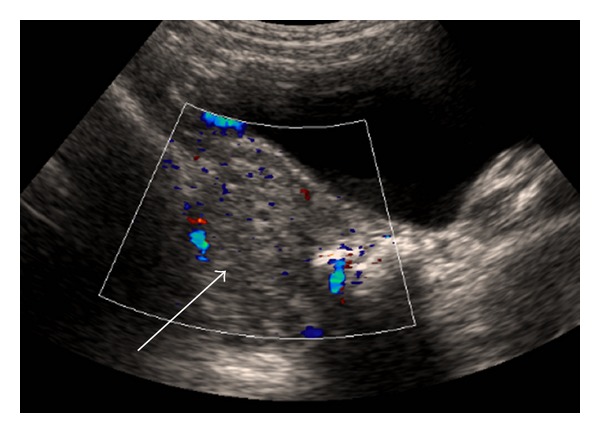
Doppler ultrasound of a large right ovarian cyst (estimated ovarian volume of 41 cm^3^) demonstrating arterial flow but scant venous outflow.

Computed tomography (CT) scan of the abdomen and pelvis with intravenous contrast demonstrated normal solid organs and normal bowel-gas pattern. A noninflammed appendix was visualized. A complex loculated fluid collection within the right adnexa and associated pelvic free fluid were seen (Figure 1). A follow-up abdominal ultrasound with Doppler, performed to further interrogate the right lower quadrant, revealed a significantly enlarged right ovary with preserved arterial flow but scant venous outflow (Figure 2). The left ovary was normal.

Exploratory laparoscopy demonstrated large right ovarian cyst (5.5 cm × 3.9 cm × 3.7 cm) with the right adnexa twisted 360°. The torsion was corrected and a right ovarian cyst drained. The patient was discharged on postoperative day 2 without further complications. Follow-up ultrasound performed three months later showed developing follicles in both adnexa with good arterial and venous blood flow.

## 3. Discussion

Ovarian torsion is a rare problem within the pediatric population, yet it represents a true gynecological and surgical emergency [1, 2]. Ovarian torsion accounts for approximately 2.7% of all cases of acute abdominal pain in children [3, 4]. Adnexal torsion is often difficult to diagnoe given the presence of nonspecific symptoms and more commonly encountered diagnoses. The presentation of adnexal torsion can mimic appendicitis, urinary tract infection, renal colic, gastroenteritis, or other conditions of acute abdominal and pelvic pain [5].

Torsion occurs more frequently in adolescents and young women [6]. For premenarchal patients, adnexal torsion occurs mostly in neonates [7]. Here, fetal and newborn ovarian cysts develop due to the high levels of circulating maternal hormones. The cysts typically resolve after birth with withdrawal of maternal hormones. In two independent sonographic studies, Orsini and coworkers [8], and Salardi et al. [9] reported the typical ovary to be solid and homogeneous in echogenicity in premenarchal girls less than six years of age. The homogeneous echogenicity is interpreted to represent ovarian parenchyma without cysts. Later, Cohen et al. [10] showed microcysts (<9 mm in greatest length) to be common in girls 2–12 years old but macrocyts to be less common, and no cyst was seen in any girl aged 2–12 in their study of the size described herein.

Torsion occurs frequently (60%) on the right side presumably because the sigmoid colon leaves limited space for adnexal movement [4, 11, 12]. The predominance of right-sided abdominal pain confounds diagnosis, and 38% of children with identified adnexal masses in one study were initially diagnosed in the emergency department with appendicitis [5].

Torsion is often associated with preexisting ovarian pathology, [6, 13] yet large cysts (as was the case in our patient) are thought to be less likely to undergo torsion secondarily to their size and mass [14]. The large size of our patient's right ovary potentially limited the torsion, which facilitated ovarian salvage as the arterial supply was not yet compromised. Normal prepubertal ovarian volume is approximately 1-2 cm^3^ [15]. Our patient had an estimated right ovarian volume of 40 cm^3^.

The diagnosis of ovarian torsion was supported by ultrasound (approximately 87% accurate for ovarian pathology [16]). While CT may be useful in diagnosing ovarian torsion [17, 18], its utility here was in discerning abdominal versus gynecological pathology, for example, CT ruled out appendicitis but did not rule in, satisfactorily ovarian torsion secondary to an ovarian cyst.

## 4. Conclusion

Prompt diagnosis and emergent surgical intervention are keys to ovary salvage, especially considering the sensitive nature of ovarian loss in the prepubescent patient. A misdiagnosis can have dire consequences including ovarian loss. Our patient was able to undergo ovarian salvage as blood flow was restored after correction. Many surgeons recommended against detorsing the ovary and prefer oophorectomy for concerns of embolization [18, 19], yet no strong evidence exists to support this claim [2, 20–22]. As demonstrated in this paper, ovarian torsion can occur at any age. Therefore a high index of suspicion coupled with radiographic evidence and clinical presentation will facilitate prompt diagnosis and ovarian salvage with significantly reduced patient comorbidity.
